# Talquetamab in Japanese patients with relapsed/refractory multiple myeloma in the MonumenTAL-1 study

**DOI:** 10.1007/s12185-025-04134-6

**Published:** 2025-12-17

**Authors:** Shigeki Ito, Yoshiaki Kuroda, Kazutaka Sunami, Kosei Matsue, Kazunori Imada, Hideto Tamura, Ei Fujikawa, Hiroshi Yamazaki, Mikihiro Takamoto, Lixia Pei, Xiang Qin, Tara J. Masterson, Michela Campagna, Veronique Vreys, Bonnie W. Lau, Yasushi Takamatsu

**Affiliations:** 1 Iwate Medical University School of Medicine, 2-1-1 Idaidori, Yahaba, Iwate 028-3695 Japan; 2 NHO, Hiroshimanishi Medical Center, Hiroshima, Japan; 3 NHO, Okayama Medical Center, Okayama, Japan; 4 Kameda Medical Center, Kamogawa, Chiba Prefecture Japan; 5 Japanese Red Cross Osaka Hospital, Osaka, Japan; 6 Dokkyo Medical University Saitama Medical Center, Saitama, Japan; 7 Johnson & Johnson, Tokyo, Japan; 8 Johnson & Johnson, Raritan, NJ USA; 9 Johnson & Johnson, Spring House, PA USA; 10 Johnson & Johnson, Madrid, Spain; 11 Johnson & Johnson, Beerse, Belgium; 12 Fukuoka University Hospital, Fukuoka, Japan

**Keywords:** Talquetamab, GPRC5D, Japanese patients, Bispecific antibody, Multiple myeloma

## Abstract

Talquetamab is the first G protein–coupled receptor family C group 5 member D (GPRC5D) × CD3 bispecific antibody approved for relapsed/refractory multiple myeloma (RRMM). We report the first efficacy and safety results of talquetamab in Japanese patients enrolled as a separate cohort in the global MonumenTAL-1 study. Between July 2022 and December 2023, 36 patients were enrolled and received 0.4 mg/kg talquetamab weekly. Median follow-up was 13.4 months. The overall response rate was 77.8% (55.6% achieved complete response or better). Median time to first response was 1.2 months. Twelve-month duration of response, progression-free survival, and overall survival rates were 66.4%, 56.3%, and 74.1%, respectively. On-target, off-tumor adverse events (AEs), including taste-, skin- (non-rash), nail-, and rash-related AEs, were common (80.6%, 66.7%, 55.6%, and 36.1%, respectively) and mostly grade 1/2. Cytokine release syndrome occurred in 75.0% of patients; all events were grade 1/2. The rate of infection was 52.8%; the rate of grade 3/4 infection was 16.7% (one led to death [pneumonia]). One patient discontinued talquetamab and three patients died due to AEs. Results were generally consistent with the global MonumenTAL-1 population, demonstrating talquetamab as an important novel treatment for Japanese patients with RRMM. Clinical trial registration number: NCT03399799/NCT04634552.

**Supplementary Information:**

The online version contains supplementary material available at 10.1007/s12185-025-04134-6.

## Introduction

Multiple myeloma (MM) is a hematologic cancer in which plasma cells proliferate excessively and overproduce abnormal immunoglobulins, leading to multiple organ system complications [1]. Globally, MM is the third most common hematologic malignancy with a 5-year prevalence of 6.8 per 100,000 individuals and approximately 121,388 deaths reported in 2022 [2]. In Asia, Japan has the highest incidence of MM, and incidence has steadily increased over time with more than 7000 new cases reported in 2019 [3]. Established therapies for the treatment of MM in Japan are generally consistent with the rest of the world and include immunomodulatory drugs (IMiDs), proteasome inhibitors (PIs), and anti-CD38 monoclonal antibodies (mAbs) [4–7]. In the relapsed/refractory setting, no consensus standard of care exists, although novel B-cell maturation antigen (BCMA)-targeting immunotherapies, including ciltacabtagene autoleucel and idecabtagene vicleucel (chimeric antigen receptor [CAR]-T cell therapies) and teclistamab and elranatamab (bispecific antibodies), have been approved in this setting in Japan [3, 8–12]. Despite these newer therapies, MM remains incurable, and patients continue to experience cycles of remission and relapse, with each remission typically shorter than the last and each successive relapse with a poorer prognosis [13–15]. To address this unmet need, additional novel treatments are needed to expand treatment options for Japanese patients with relapsed/refractory MM (RRMM).

Talquetamab is a first-in-class, off-the-shelf, T-cell redirecting bispecific antibody that targets G protein–coupled receptor family C group 5 member D (GPRC5D) on myeloma cells and CD3 on T cells [16, 17]. GPRC5D is an orphan receptor with no recognized ligand and is highly expressed in malignant plasma cells but rarely in normal plasma cells [18]. Talquetamab induces T-cell–mediated cytotoxicity by bringing activated CD3-expressing T cells into close proximity to GPRC5D-expressing myeloma cells, leading to T-cell activation and subsequent lysis of myeloma cells [16–18].

Talquetamab has been approved in both the United States and Europe for patients with triple-class exposed (to an IMiD, PI, and anti-CD38 mAb) RRMM based on results from the global phase 1/2 MonumenTAL-1 study (NCT03399799/NCT04636552) [16, 17]. In MonumenTAL-1, talquetamab demonstrated high response rates of ≥ 69% in patients with triple-class exposed RRMM who received two recommended phase 2 doses (RP2Ds) of subcutaneous (SC) talquetamab: 0.4 mg/kg weekly (QW) or 0.8 mg/kg every other week (Q2W). Responses were rapid and durable: time to response (TTR) was 1.2 (QW) and 1.3 (Q2W) months, median duration of response (DOR) was 9.5 (QW) and 16.9 (Q2W) months, and 12-month overall survival (OS) rates were 76.4% (QW) and 76.8% (Q2W) [19]. Common adverse events (AEs) included cytokine release syndrome (CRS) and on-target, off-tumor (GPRC5D-related) AEs; however, talquetamab was generally tolerable and led to few treatment discontinuations (4.9% [QW] and 9.1% [Q2W]) [17, 19].

A phase 1 analysis of talquetamab in 15 Japanese patients receiving three doses of talquetamab (0.135 mg/kg QW, 0.4 mg/kg QW, and 0.8 mg/kg Q2W) revealed no dose-limiting toxicities, deaths, or dose reductions/treatment discontinuations due to AEs. Common AEs included neutropenia, lymphopenia, CRS, and GPRC5D-related AEs, similar to the global MonumenTAL-1 study, and overall response rate (ORR) was 60.0% (0.4 mg/kg QW) and 83.3% (0.8 mg/kg Q2W) [19, 20]. Here, we report outcomes with talquetamab 0.4 mg/kg QW in Japanese patients enrolled in a separate phase 2 cohort of the MonumenTAL-1 study.

## Materials and methods

### Study design and patients

The methodology of MonumenTAL-1 was previously published [19, 21]. Briefly, MonumenTAL-1 is a first-in-human, phase 1/2, open-label, multicenter study of talquetamab monotherapy in patients with RRMM. The phase 1 dose escalation and expansion portion of the study identified two RP2Ds: SC talquetamab 0.4 mg/kg QW and 0.8 mg/kg Q2W [21]. In phase 2, efficacy and safety were further assessed in patients receiving the RP2Ds of talquetamab. Eligible patients in phase 2 were aged ≥ 18 years, had measurable MM per International Myeloma Working Group (IMWG) criteria [22], had ≥ 3 prior lines of therapy (≥ 1 PI, ≥ 1 IMiD, and ≥ 1 anti-CD38 mAb), had documented progressive disease based on IMWG 2016 criteria, and had an Eastern Cooperative Oncology Group performance status ≤ 2. Patients enrolled in the global cohorts included those who were naive or exposed to prior T-cell redirection therapies, such as CAR-T and bispecific antibodies; prior exposure to antibody–drug conjugates was permitted. Patients from Japan with no prior exposure to T-cell redirection therapies were enrolled in a separate phase 2 cohort of MonumenTAL-1, referred to as the Japan cohort.

This study was conducted in accordance with the principles of the Declaration of Helsinki and Good Clinical Practice guidelines of the International Council for Harmonisation. Study protocol and amendments were approved by the institutional review board at each site, and all authors affirm that the study was conducted in accordance with the protocol. All patients provided written informed consent.

### Treatment

Patients in the Japan cohort received SC talquetamab 0.4 mg/kg QW. To mitigate the risk of CRS, patients received two step-up doses (0.01 mg/kg and 0.06 mg/kg) of talquetamab and pretreatment medications (glucocorticoid, antihistamine, and antipyretic) prior to step-up doses and initial full treatment dose. Hospitalization for ≥ 48 h was required from the start of each step-up dose and first full treatment dose. Patients received treatment until disease progression, unacceptable toxicity, withdrawal of consent, or end of study.

### Endpoints and assessments

The primary endpoint for the Japan cohort was ORR. Key secondary endpoints included DOR, rate of very good partial response or better (≥ VGPR), rate of complete response or better (≥ CR), rate of stringent complete reponse (sCR), TTR, progression-free survival (PFS), OS, incidence of AEs, and pharmacokinetics (PK).

ORR was defined as the proportion of patients who achieved a partial response or better according to IMWG 2016 consensus criteria [22]. Disease response and progression were assessed by an independent review committee. CRS and immune effector cell–associated neurotoxicity syndrome (ICANS) were graded by American Society for Transplantation and Cellular Therapy criteria [23]. All other AEs were graded by Common Terminology Criteria for Adverse Events (CTCAE) v4.03. Serial PK serum samples were collected from Cycle 1 until Cycle 13 and at the end of treatment. Serum concentrations of talquetamab were analyzed using a validated, specific, and sensitive immunoassay method with a lower limit of qualification of 0.5 ng/mL.

Efficacy and safety analyses were based on a data cutoff date of September 2024. PK analyses were based on a data cutoff date of May 2024.

### Statistical analyses

Efficacy and safety data were analyzed in Japanese patients who received at least one dose of talquetamab. ORR and 95% exact confidence intervals (CIs) were calculated; statistical significance was considered achieved if the lower limit of the two-sided 95% CI of the ORR was > 30%. The Kaplan–Meier method was used to analyze DOR, PFS, and OS. Data were summarized using descriptive statistics.

## Results

### Patients

Between July 2022 and December 2023, 36 patients were enrolled in the talquetamab 0.4 mg/kg QW Japan cohort. The median age was 70.5 years, and the majority of patients were male (55.6%). Patients had a median of 3.5 prior lines of therapy (range 3–13). Extramedullary disease, defined as soft tissue plasmacytomas not associated with bone, was present in two (5.6%) patients. Thirteen (39.4%) patients had high-risk cytogenetics, defined as the presence of del(17p), t(4;14), and/or t(14;16), consistent with the 2015 Revised International Staging System [24]. Most patients were triple-class refractory (25 [69.4%]); eight (22.2%) were penta-drug refractory and 35 (97.2%) were refractory to the last line of therapy (Table 1).

**Table 1:** Patient demographics and baseline characteristics

Characteristic	Talquetamab 0.4 mg/kg SC QW^a^ (N = 36)
Median age, years (range)	70.5 (46–81)
Male, n (%)	20 (55.6)
High-risk cytogenetics,^b^ n (%)	13 (39.4)
ISS stage, n (%)	
I	18 (50.0)
II	14 (38.9)
III	4 (11.1)
Extramedullary plasmacytomas,^c^ n (%)	2 (5.6)
Bone marrow plasma cells ≥ 60%,^d^ n (%)	2 (5.7)
Baseline ECOG PS, n (%)	
0	24 (66.7)
1	9 (25.0)
2	3 (8.3)
Prior ASCT, n (%)	22 (61.1)
Median time from diagnosis to first dose, years (range)	5.31 (0.6–20.3)
Median prior LOT (range)	3.5 (3–13)
Exposure status, n (%)	
Triple-class^e^	36 (100.0)
Penta-drug^f^	21 (58.3)
Refractory status, n (%)	
Triple-class^e^	25 (69.4)
Penta-drug^f^	8 (22.2)
PI	26 (72.2)
IMiD	33 (91.7)
Thalidomide	1 (2.8)
Lenalidomide	25 (69.4)
Pomalidomide	23 (63.9)
Anti-CD38 mAb	34 (94.4)
To last LOT	35 (97.2)

*ASCT* autologous stem cell transplant, *ECOG PS* Eastern Cooperative Oncology Group performance status, *IMiD* immunomodulatory drug, *ISS* International Staging System, *LOT* line of therapy, *mAb* monoclonal antibody, *PI* proteasome inhibitor, *QW* weekly, *SC* subcutaneous

^a^With 2 step-up doses

^b^del(17p), t(4;14), and/or t(14;16); percentage calculated from n = 33

^c^Soft tissue plasmacytomas not associated with the bone were included

^d^Percentage calculated from n = 35; maximum value from bone marrow biopsy or bone marrow aspirate is selected if both results are available

^e^≥ 1 PI, ≥ 1 IMiD, and ≥ 1 anti-CD38 mAb

^f^≥ 2 PIs, ≥ 2 IMiDs, and ≥ 1 anti–CD38 mAb

### Efficacy

**Fig. 1:**
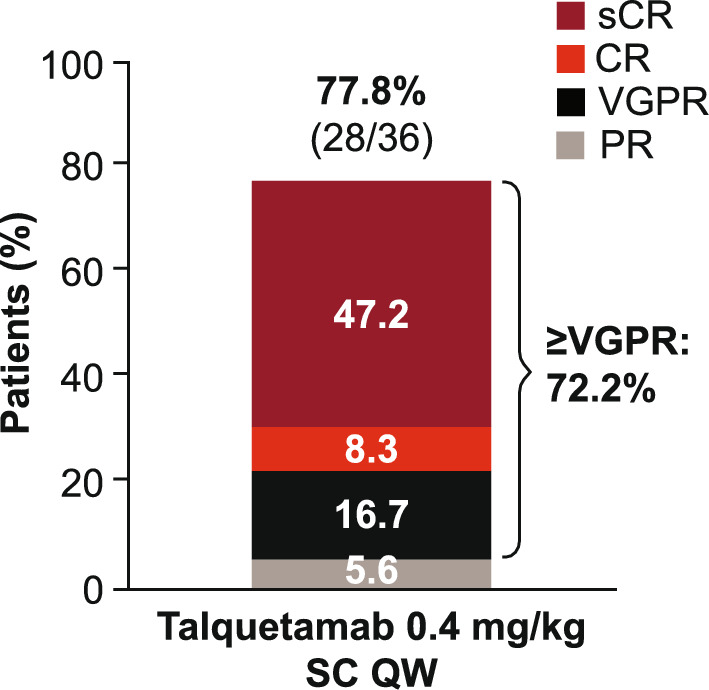
ORR (ORR was defined as sCR + CR + VGPR + PR. Due to rounding, individual response rates may not sum to the ORR. ORR was assessed by independent review committee based on International Myeloma Working Group 2016 consensus criteria in all treated patients.). Abbreviations: CR, complete response; ORR, overall response rate; PR, partial response; QW, weekly; SC, subcutaneous; sCR, stringent complete response; VGPR, very good partial response

As of September 2024, median follow-up was 13.4 (range 0.6–24.7) months. ORR (95% CI) was 77.8% (60.8–89.9) with 72.2% (54.8–85.8) of patients achieving ≥ VGPR, 55.6% (38.1–72.1) achieving ≥ CR, and 47.2% (30.4–64.5) achieving sCR (Fig. 1).

**Fig. 2:**
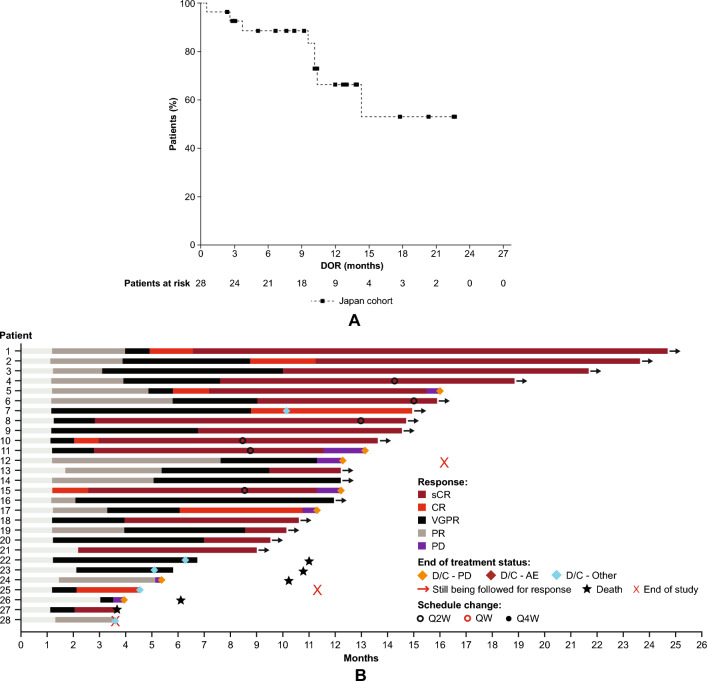
DOR (Disease response and progression were assessed by independent review committee.) (**A**) and treatment response over time in patients who had ≥ PR (**B**). Abbreviations: AE, adverse event; CR, complete response; D/C, discontinued; DOR, duration of response; PD, progressive disease; PR, partial response; Q2W, every other week; Q4W, every 4 weeks; QW, every week; sCR, stringent complete response; VGPR, very good partial response

Among the 28 responders to talquetamab, the median (range) time to first response was 1.2 (1.1–3.0) months, the median time to best response was 4.5 (1.2–11.2) months, and the median time to ≥ VGPR was 2.1 (1.1–7.6) months. The median DOR was not reached (NR) (95% CI 10.2–not estimable [NE]) with 71.4% of patients censored (Fig. 2a). Responses deepened over time (Fig. 2b). The 9-month DOR rate (95% CI) was 88.7% (68.8–96.2), and the 12-month DOR rate was 66.4% (40.9–82.9).

**Fig. 3:**
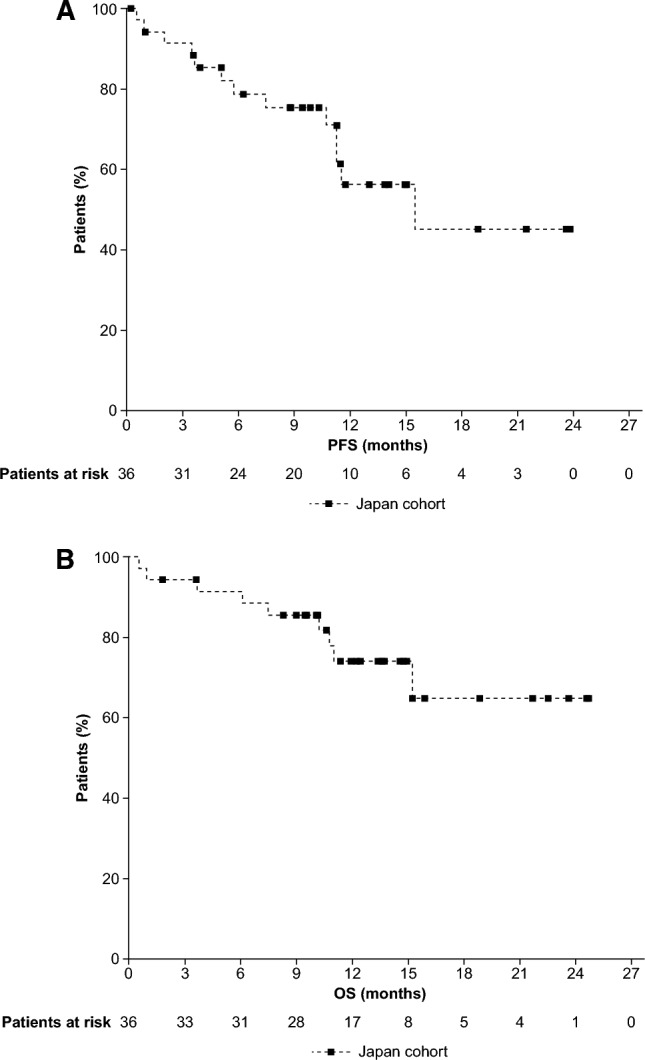
PFS (Disease response and progression were assessed by independent review committee.) (**A**) and OS (**B**). Abbreviations: OS, overall survival*;* PFS, progression-free survival

The median PFS was 15.5 (95% CI 11.3–NE) months with 63.9% of patients censored (Fig. 3a). The 9-month PFS rate (95% CI) was 75.4% (56.5–86.9), and the 12-month PFS rate was 56.3% (34.8–73.2). The median OS was NR (95% CI 15.2–NE) with 75.0% of patients censored (Fig. 3b). The 9-month OS rate (95% CI) was 85.6% (68.8–93.7), and the 12-month OS rate was 74.1% (54.3–86.3).

### Safety

The most common AEs in the Japan cohort were taste-related AEs, CRS, and skin-related (non-rash) AEs. The most common grade 3/4 AEs were hematologic toxicities (Table 2).

**Table 2:** Hematologic and non-hematologic adverse events

AEs, n (%)	Talquetamab 0.4 mg/kg SC QW^a^ (N = 36)	
	Any grade	Grade 3/4
Hematologic AEs		
Lymphopenia	17 (47.2)	17 (47.2)
Neutropenia	14 (38.9)	10 (27.8)
Anemia	10 (27.8)	8 (22.2)
Thrombocytopenia	9 (25.0)	7 (19.4)
Nonhematologic AEs		
Taste-related^b,c^	29 (80.6)	NA
CRS	27 (75.0)	0
Skin-related (non-rash)^d^	24 (66.7)	0
Nail-related^e^	20 (55.6)	0
Infections^f^	19 (52.8)	6 (16.7)^g^
Pyrexia	17 (47.2)	1 (2.8)
Rash-related^h^	13 (36.1)	1 (2.8)
C-reactive protein increased	11 (30.6)	0
Weight decreased	8 (22.2)	3 (8.3)
Nausea	8 (22.2)	0
Dry mouth	7 (19.4)	0
Injection site erythema	7 (19.4)	0
Decreased appetite	6 (16.7)	0
Dysphagia	6 (16.7)	0
Malaise	6 (16.7)	0
Lipase increased	5 (13.9)	4 (11.1)
Arthralgia	5 (13.9)	1 (2.8)
Back pain	5 (13.9)	1 (2.8)
Oropharyngeal pain	5 (13.9)	0
Vomiting	5 (13.9)	0
Hypokalemia	4 (11.1)	4 (11.1)
Diarrhea	4 (11.1)	1 (2.8)
Diplopia	4 (11.1)	0
Headache	4 (11.1)	0
Stomatitis	4 (11.1)	0

Note: AEs listed are those occurring in ≥ 10% of patients

*AE* adverse event, *CRS* cytokine release syndrome, *NA* not applicable, *QW* weekly, *SC* subcutaneous

^a^With 2 step-up doses

^b^Includes dysgeusia, ageusia, hypogeusia, and taste disorder

^c^Per Common Terminology Criteria for Adverse Events, the maximum grade for these events is 2

^d^Includes skin exfoliation, dry skin, pruritus, and palmar-plantar erythrodysesthesia syndrome

^e^Includes nail discoloration, nail disorder, onycholysis, onychomadesis, onychoclasis, nail dystrophy, nail toxicity, and nail ridging

^f^Infections are reported at the System Organ Class level

^g^One grade 3/4 infection led to death (pneumonia)

^h^Includes rash, maculopapular rash, erythematous rash, and erythema

CRS occurred in 75.0% of patients (Table 2). All events were grade 1 (58.3%) or 2 (16.7%) and were largely confined to step-up and Cycle 1 Day 1 doses, with few events occurring on or after Cycle 2 (8.3%). Median time to onset of CRS was 30.5 h, and median duration of CRS was 12.5 h (Supplementary Table 1). Supportive measures were given to 26 (72.2%) patients and included tocilizumab (61.1%), acetaminophen (44.4%), and intravenous fluids (25.0%). Recurrent CRS events occurred in 36.1% of patients, were all grade 1/2, and mostly occurred during step-up and Cycle 1 Day 1 doses. A small proportion of patients (5.6%) had a worse grade during any subsequent CRS event. Median time to onset of recurrent CRS was 30.9 h, and median duration of recurrent CRS was 12.3 h. Cycle delays or dose modifications due to CRS occurred in six (16.7%) patients. All CRS events resolved, and no patients discontinued treatment due to CRS.

No patients reported an ICANS event. The only neurotoxicity event was a grade 1 headache.

**Fig. 4:**
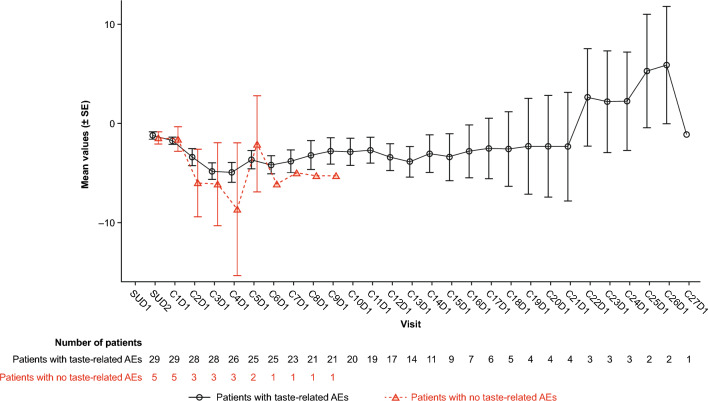
Mean weight percentage change from baseline over time for patients with and without taste-related AEs. Abbreviations: AE, adverse event; C, cycle; D, day; SUD, step-up dose; SE, standard error

On-target, off-tumor (GPRC5D-related) AEs included taste-, skin- (non-rash), nail-, and rash-related AEs. Taste-related AEs were the most common AE reported with talquetamab (80.6%) (Table 2). Taste-related AEs were reported as a grouped term that included dysgeusia, ageusia, hypogeusia, and taste disorder, which all have a maximum grade of 2 per CTCAE. Median time to onset of taste-related AEs was 11 days, and median duration was 195 days (Supplementary Table 2). More detailed information on taste-related AEs, including the specific toxicity and patient experience of the toxicity, is shown in Table 3. Dose modifications (i.e., dose delays, skips, or reductions) due to taste-related AEs occurred in one (2.8%) patient; 31% of events resolved at the time of data cutoff, and no patients discontinued treatment due to taste-related AEs. Weight decrease was reported in eight (22.2%) patients; of these, three (8.3%) had grade 3 events. Following talquetamab treatment, weight decreased until Cycle 4, then began to stabilize. This trend was similar irrespective of whether patients had taste-related events, although numbers are small (Fig. 4).

**Table 3:** Taste-related AEs in ten patients^a^ receiving talquetamab

Patient	AE name (preferred term/reported term)	Onset (days)^b^	Grade	Reported symptoms
1	Dysgeusia/dysgeusia	12	1	No taste of saltiness or flavor
2	Dysgeusia/dysgeusia	10	1	No taste of saltiness or flavor
3	Dysgeusia/dysgeusia	13 22	1 2	Ageusia (loss of taste) Cacogeusia (unpleasant taste); degree and symptoms vary day to day
4	Dysgeusia/dysgeusia	5 5	1 1	First, hypogeusia (diminished saltiness and sweetness) was reported Later, dissociated dysgeusia (loss of saltiness and sweetness) was reported Tingling sensation in the oral cavity; no stomatitis observed
5	Dysgeusia/dysgeusia	9	2	Ageusia
6	Dysgeusia/worsening of dysgeusia	15	2	The patient had grade 1 dysgeusia due to radiotherapy as medical history After study treatment, grade 2 worsening of dysgeusia was reported First, hypogeusia was reported. Then, ageusia was reported
7	Dysgeusia/dysgeusia	9	2	First, dissociated dysgeusia (loss of sweetness and pungency) was reported Later, hypogeusia was reported
8	Dysgeusia/dysgeusia	10	1	Hypogeusia (diminished sweetness)
9	Dysgeusia/heterogeusia	164 332	2 1	First, dissociated dysgeusia (loss of sweetness) was reported Second, heterogeusia (sweetness, water tastes bitter) was reported Third, heterogeusia (wholly) was reported Fourth, small improvement in heterogeusia (sweetness) was reported Improvement in tasting flavors was reported Later, hypogeusia (diminished sweetness and saltiness) was reported
10	Dysgeusia/dysgeusia	9	2	First, ageusia was reported Later, ageusia (excluding sweetness) was reported

*AE* adverse event

^a^In patients who provided comments of their symptoms

^b^Day of AE onset relative to initial step-up dose

**Fig. 5:**
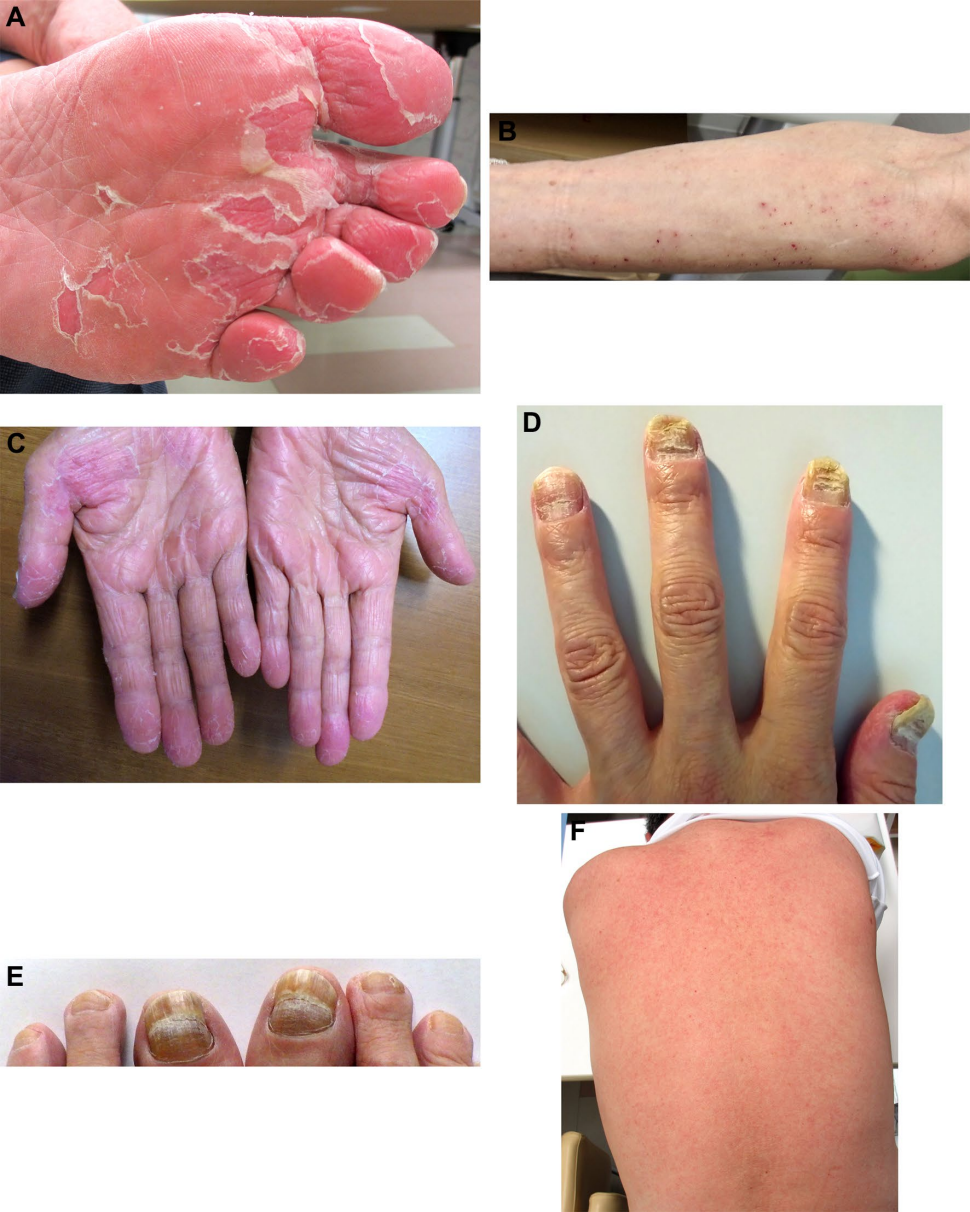
Images (Written consent for the publication of these medical photographs was obtained from the patient(s).) of dermatologic GPRC5D-related AEs due to talquetamab, including skin-related (non-rash) AEs (**A–C**), nail-related AEs (**D, E**), and rash-related AEs (**F**). Abbreviations: AE adverse event; GPRC5D, G protein-coupled receptor class 5 member D

Skin-related (non-rash) AEs were reported as a grouped term that included skin exfoliation, dry skin, pruritus, and palmar-plantar erythrodysesthesia syndrome (Fig. 5a–c), and occurred in 66.7% of patients (Table 2). All events were low grade (1/2). Median time to onset of skin-related (non-rash) AEs was 21 days, and median duration was 46 days (Supplementary Table 2). Dose modifications due to skin-related (non-rash) AEs occurred in two (5.6%) patients; 42.1% of events resolved at the time of data cutoff, and no patients discontinued treatment due to skin-related (non-rash) AEs.

Nail-related AEs were reported as a grouped term that included nail discoloration, nail disorder, onycholysis, onychomadesis, onychoclasis, nail dystrophy, nail toxicity, and nail ridging (Fig. 5d and e), and occurred in 55.6% of patients (Table 2). All were low grade (1/2). Median time to onset of nail-related AEs was 51 days, and median duration was 109 days (Supplementary Table 2). No dose modifications were required for nail-related AEs; 33.3% of events were resolved at the time of data cutoff, and no patients discontinued treatment due to nail-related AEs.

Rash-related AEs were reported as a grouped term that included rash, maculopapular rash, erythematous rash, and erythema (Fig. 5f), and occurred in 36.1% of patients (Table 2). The majority were grade 1/2. Median time to onset of rash-related AEs was 15 days, and median duration was 36 days (Supplementary Table 2). Dose modifications due to rash-related AEs occurred in two (5.6%) patients; most events resolved (88.9%), and no patients discontinued treatment due to rash-related AEs.

Hematologic AEs occurring in ≥ 25% of patients included lymphopenia (47.2%), neutropenia (38.9%), anemia (27.8%), and thrombocytopenia (25.0%). The most common grade 3/4 hematologic AEs were lymphopenia (47.2%) and neutropenia (27.8%) (Table 2).

**Fig. 6:**
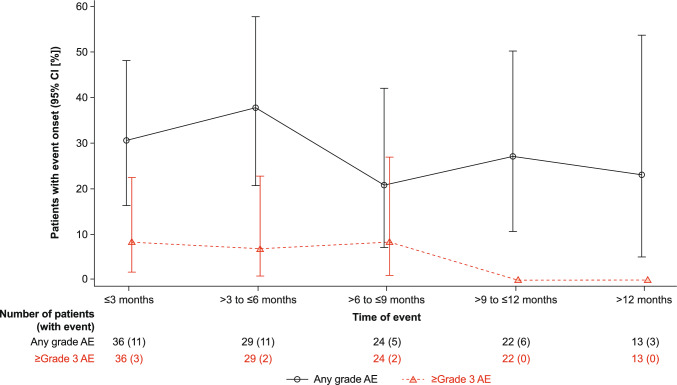
New onset any grade and grade ≥ 3 infections over time. Abbreviations: AE, adverse event; CI, confidence interval

Infections occurred in 19 (52.8%) patients; pneumonia and upper respiratory tract infections were the most common. Grade 3/4 infections occurred in six (16.7%) patients (Table 2) (one of which resulted in death [grade 5 pneumonia]); excluding the grade 5 infection, grade 3/4 infections included pneumonia (two patients; 5.6%), otitis media, sepsis, and staphylococcal sepsis (one patient each; 2.8% each). Three (8.9%) patients had COVID-19; all were grade 1/2. Grade ≥ 3 infections generally occurred in the first 6–9 months of treatment and then declined thereafter (Fig. 6). Opportunistic infections occurred in one patient (grade 2 cytomegalovirus infection); the infection resolved and talquetamab was continued without any dose modifications. Hypogammaglobulinemia was reported in three (8.3%) patients.

Fifteen (41.7%) patients reported serious AEs, seven (19.4%) of which were considered by the investigator as related to talquetamab. Grade 5 AEs occurred in three (8.3%) patients; of these, two patients had respiratory failure (not attributed to talquetamab) and one patient had interstitial lung disease (not attributed to talquetamab) and pneumonia (attributed to talquetamab). There were no dose reductions due to AEs. AEs resulted in treatment discontinuation in one (2.8%) patient (staphylococcal sepsis).

### Pharmacokinetics

The mean trough concentration (C_trough_) was higher than the 90% maximal effective concentration values identified in an ex vivo cytotoxicity assay from first target dose.

## Discussion

In this phase 2 analysis of Japanese patients with triple-class exposed RRMM receiving the novel agent talquetamab in the MonumenTAL-1 study, a high ORR (77.8%) was observed, and responses were deep (72.2% ≥ VGPR; 55.6% ≥ CR) and durable (median DOR was NR, 12-month DOR rate was 66.4%, median PFS was 15.5 months, and 12-month PFS rate was 56.3%). Efficacy outcomes in the Japan cohort were generally consistent with the global MonumenTAL-1 QW cohort [19]. These data are impressive considering the current treatment landscape for patients with RRMM, in which no consensus standard of care exists [25]. This is underscored by a recent real-world analysis that identified more than 100 unique regimens used in the treatment of patients with triple-class exposed RRMM who experienced poor treatment outcomes (ORR 31.8%; median PFS 4.6 months) [26]. In indirect treatment comparisons utilizing the global MonumenTAL-1 cohorts, talquetamab has demonstrated superior efficacy compared with standard treatment regimens (real-world physician’s choice) in triple-class exposed RRMM, further supporting talquetamab as a compelling treatment in this setting [27, 28].

Similar to the global MonumenTAL-1 population, CRS was one of the most commonly reported AEs in the Japan cohort. CRS is characterized by systemic immune activation, including excessive elevation of cytokines and inflammatory processes that can be triggered by infections or T-cell redirecting therapies, such as CAR-T and bispecific antibodies. Symptoms of CRS range from low-grade fever to serious multi-organ failure and may affect patient outcomes and quality of life [23, 29]. Generally, CRS is less frequent, less severe, has an earlier onset, and has a shorter duration in patients treated with bispecific antibodies compared with CAR-T therapies [29–32]. In the Japan cohort, CRS events generally occurred early during step-up and Cycle 1 Day 1 doses, were all low grade (1/2), and did not result in treatment discontinuations; these data closely align with the global MonumenTAL-1 cohorts and are consistent with other T-cell redirecting bispecific antibodies in RRMM [33, 34]. Although guidance exists to manage CRS [35], additional strategies to mitigate CRS are being investigated, including prophylactic tocilizumab use and alternative step-up dosing schedules [36, 37]. In a small cohort of patients (N = 12) from the global MonumenTAL-1 study who received prophylactic tocilizumab, incidence and severity of CRS were reduced without impacting antimyeloma activity [37]. Although preliminary, this strategy may potentially facilitate outpatient administration of talquetamab, thereby improving both safety and convenience of talquetamab, reducing burden of hospitalization, and elevating patient experience.

GPRC5D-related AEs, including taste-, skin- (non-rash), nail- and rash-related AEs, are common with talquetamab and have previously been described in the global MonumenTAL-1 cohorts [19, 38]. These on-target, off-tumor AEs may be related to the expression pattern of GPRC5D, which has been found in epithelial tissues, including hair, skin cells associated with hair follicle-specific keratin, eccrine glands, and filiform papillae on the tongue. GPRC5D expression is also believed to occur in the nailbed of humans due to expression reported in the nailbeds of mice [18, 39–42].

Similar to the global MonumenTAL-1 cohorts, GPRC5D-related AEs in the Japan cohort were common and mostly low grade; none resulted in treatment discontinuation, and few dose modifications were required. The most common treatments used to manage taste-related AEs in the Japan cohort were consistent with those in the global cohorts and included anti-infective, anti-inflammatory, and corticosteroid oral medications (Supplementary Table 2). In the global MonumenTAL-1 study, dose modifications were implemented in 7.7% of patients across cohorts and based on investigator experience, they appear to be the most effective strategy in managing taste-related AEs [38]. In the Japan cohort, dose modifications were also implemented in 1 (2.8%) patient to manage taste-related AEs. The novelty and subjectivity of taste-related AEs with talquetamab, as well as limitations of CTCAE grading that defines a maximum severity as 2, have posed challenges to identifying and managing taste-related AEs [43]. To contribute to our understanding, we captured the taste disorder experience of ten patients in the Japan cohort (Table 3), which highlighted the variability of each patient’s experience. A recently initiated study, TALISMAN, is assessing strategies to mitigate and manage taste-related toxicities due to talquetamab, and will provide needed data on taste-related assessment tools as well as the impact of taste-related AEs on patient experience [44].

Taste-related toxicities may have an impact on diet given the potential change to patients’ experience of food taste or food interest, and thus an impact on weight. However, preliminary investigation into the association of weight loss with taste-related toxicities in the Japan cohort indicated that weight loss occurred regardless of whether patients experienced taste-related AEs. This outcome is similar to what has been observed in the global MonumenTAL-1 cohorts, which assessed weight loss in association with a broader group of oral toxicities (including taste-related toxicities) [45]. Studies are ongoing to further characterize and understand weight loss with talquetamab. Early referral to a nutritionist should be considered to provide guidance on maintaining a balanced diet and weight with talquetamab [38].

In terms of dermatologic GPRC5D-related AEs, the incidence, severity, timing, and dose modifications for skin- (non-rash), nail-, and rash-related AEs were similar between the Japan cohort and the global MonumenTAL-1 cohorts. Management of dermatologic GPRC5D-related AEs largely mirrored that of the global population, with common approaches including use of topical steroids, petroleum based moisturizers, and oral antihistamines (Supplementary Table 2). Topical heparin, mucopolysaccharide polysulfate cream, and crotamiton were additionally used in the Japan cohort due to variations in product availability and institutional protocols. Images of dermatologic GPRC5D-related AEs in the Japan cohort are presented here (Fig. 5) and may help clinicians to identify these toxicities promptly. Investigation in the global MonumenTAL-1 population has indicated that although dermatologic GPRC5D-related AEs may impact quality of life, they are typically mild and generally resolve, with the exception of nail-related toxicities due to slower growth of nails [38].

In the Japan cohort, the incidence and timing of infections (generally restricted to the first 6 months of treatment) were comparable to the global MonumenTAL-1 population. Of note, like the global MonumenTAL-1 population, the rate of grade 3/4 infections (16.7%) was lower in the Japan cohort than rates observed in published studies of BCMA-targeting bispecific antibodies (40‒55%) [19, 33, 46]. Unlike BCMA, GPRC5D expression is restricted to malignant plasma cells with limited expression on other hematopoietic cells, including cells across the continuum of the B-cell lineage [18, 41, 42]. We have previously shown no decreases in B-cell counts or polyclonal immunoglobulin G concentration following talquetamab treatment in the global MonumenTAL-1 study [47]. Together, these data continue to support talquetamab as a B-cell–sparing treatment that allows for preservation of humoral immune function to permit better control of infections [47].

There are some limitations to this study, including the single-arm study design and relatively small sample size of the Japan cohort. Although the Japan cohort had a relatively short follow-up period, data collection is ongoing. While the 0.8 mg/kg Q2W dose was not studied in this analysis, the safety and efficacy of the Q2W dose were assessed in a separate Japan phase 1 study (n = 6), and response was observed in five of six Japanese patients [20].

In conclusion, efficacy and safety results with talquetamab among Japanese patients with triple-class exposed RRMM who participated in the MonumenTAL-1 study were generally consistent with the global MonumenTAL-1 population [19]: talquetamab demonstrated rapid, deep, and durable responses, with low rates of discontinuations due to AEs, none of which were due to on-target, off-tumor AEs. Overall, these data support use of talquetamab as a novel immunotherapy for Japanese patients with triple-class exposed RRMM.

## Supplementary Information

Below is the link to the electronic supplementary material.

Supplementary file1 (DOCX 76 KB)

## Data Availability

The data sharing policy of Johnson & Johnson is available at https://www.jnj.com/innovativemedicine/node/87. As noted on this site, requests for access to the study data can be submitted through Yale Open Data Access (YODA) Project site at http://yoda.yale.edu.
