# Supplementary material for: Talquetamab in Japanese patients with relapsed/refractory multiple myeloma in the MonumenTAL-1 study
Source: Int J Hematol. 2025 Dec 17;123(4):580–92. doi: 10.1007/s12185-025-04134-6 (PMC13083461; doi:10.1007/s12185-025-04134-6)
Supplement: Supplementary file 1 — Supplementary file1 (DOCX 76 KB) [file 12185_2025_4134_MOESM1_ESM.docx]

# Supplementary Information

## Supplementary Table 1 Incidence, symptoms, timing, recurrence, and supportive measures given for CRS

|  | Talquetamab 0.4 mg/kg SC QW^a^  (N = 36) |
| --- | --- |
| Patients with CRS, n (%) | 27 (75.0) |
| Grade 1 | 21 (58.3) |
| Grade 2 | 6 (16.7) |
| Grade 3 | 0 |
| Grade 4 | 0 |
| Grade 5 | 0 |
| Symptoms of CRS (>10%), n (%) |  |
| Pyrexia | 27 (75.0) |
| Hypotension | 4 (11.1) |
| Median time to onset, hours (range)^b^ | 30.5 (16.0–72.6) |
| Median duration, hours (range)^c^ | 12.5 (1.0–112.6) |
| Patients with CRS up to 1^st^ full dose, n (%) |  |
| 1^st^ step-up dose | 14 (38.9) |
| 2^nd^ step-up dose | 13 (36.1) |
| 1^st^ full dose | 10 (27.8) |
| Patients with CRS cycle 2+, n (%) | 3 (8.3) |
| Patients receiving supportive measures, n (%)^d^ | 26 (72.2) |
| Tocilizumab^e^ | 22 (61.1) |
| Acetaminophen | 16 (44.4) |
| IV fluids | 9 (25.0) |
| Corticosteroids | 2 (5.6) |
| Oxygen | 2 (5.6) |
| Nasal cannula low flow (≤6 L/min) | 2 (5.6) |
| Other | 5 (13.9) |
| Patients with >1 CRS event, n (%) | 13 (36.1) |
| Grade worsened at subsequent event | 2 (5.6) |

*CRS* cytokine release syndrome; *IV* intravenous; *QW* weekly; *SC* subcutaneous.
^a^With 2–3 step-up doses. ^b^Relative to the most recent dose. ^c^Includes CRS with both start and end dates available. ^d^Patients could receive more than one supportive therapy. ^e^Tocilizumab was advised for grade 2 and higher but allowed at grade 1; the protocol did not recommend prophylactic tocilizumab use.

## Supplementary Table 2 Incidence, timing, outcomes, dose modifications needed, and supportive measures given for taste-, skin- (non-rash), nail-, and rash-related AEs

|  | Talquetamab 0.4 mg/kg SC QW^a^  (N = 36) |
| --- | --- |
| Taste-related AE^b^ |  |
| Total, n (%) | 29 (80.6) |
| Leading to dose modification, n (%) | 1 (2.8) |
| Onset, days, median (range)^c^ | 11 (3–30) |
| Duration, days, median (range)^d^ | 195 (119–412) |
| Outcome, n (%) |  |
| Events, n | 29 |
| Recovered or resolved | 9 (31.0) |
| Not recovered or not resolved | 18 (62.1) |
| Recovered or resolved with sequelae | 0 |
| Recovering or resolving | 0 |
| Unknown | 0 |
| Missing | 2 (6.9) |
| Patients receiving supportive measures, n (%)^e^ | 13 (36.1) |
| Anti-inflammatory gargle solution | 4 (11.1) |
| Anti-bacterial zinc-containing mouthwash | 3 (8.3) |
| Oral beclomethasone | 2 (5.6) |
| Sodium bicarbonate mouthwash | 2 (5.6) |
| Mineral support | 2 (5.6) |
| Vitamin support | 2 (5.6) |
| Zinc lozenges | 2 (5.6) |
| Carbohydrates | 2 (5.6) |
| Skin-related (non-rash) AE^f^ |  |
| Total, n (%) | 24 (66.7) |
| Leading to dose modification, n (%) | 2 (5.6) |
| Onset, days, median (range)^c^ | 21 (6–409) |
| Duration, days, median (range)^d^ | 46 (31–311) |
| Outcome, n (%) |  |
| Events, n | 38 |
| Recovered or resolved | 16 (42.1) |
| Not recovered or not resolved | 16 (42.1) |
| Recovered or resolved with sequelae | 0 |
| Recovering or resolving | 1 (2.6) |
| Unknown | 0 |
| Missing | 5 (13.2) |
| Patients receiving supportive measures, n (%)^e^ | 22 (61.1) |
| Topical |  |
| Heparin | 14 (38.9) |
| Mucopolysaccharide polysulfate cream | 7 (19.4) |
| Diphenhydramine | 7 (19.4) |
| Hydrocortisone | 7 (19.4) |
| White petroleum jelly | 4 (11.1) |
| Betamethasone | 4 (11.1) |
| Clobetasol | 2 (5.6) |
| Crotamiton | 2 (5.6) |
| Oral |  |
| Fexofenadine | 6 (16.7) |
| Nail-related AE^g^ |  |
| Total, n (%) | 20 (55.6) |
| Leading to dose modification, n (%) | 0 |
| Onset, days, median (range)^c^ | 51 (13–203) |
| Duration, days, median (range)^d^ | 109 (8–267) |
| Outcome, n (%) |  |
| Events, n | 21 |
| Recovered or resolved | 7 (33.3) |
| Not recovered or not resolved | 12 (57.1) |
| Recovered or resolved with sequelae | 0 |
| Recovering or resolving | 0 |
| Unknown | 0 |
| Missing | 2 (9.5) |
| Patients receiving supportive measures, n (%)^e^ | 3 (8.3) |
| Topical heparin | 2 (5.6) |
| Rash-related AE^h^ |  |
| Total, n (%) | 13 (36.1) |
| Leading to dose modification, n (%) | 1 (2.8) |
| Onset, days, median (range)^c^ | 15 (1–282) |
| Duration, days, median (range)^d^ | 36 (6–260) |
| Outcome, n (%) |  |
| Events, n | 18 |
| Recovered or resolved | 16 (88.9) |
| Not recovered or not resolved | 2 (11.1) |
| Recovered or resolved with sequelae | 0 |
| Recovering or resolving | 0 |
| Unknown | 0 |
| Missing | 0 |
| Patients receiving supportive measures, n (%)^e^ | 12 (33.3) |
| Topical steroids | 5 (13.9) |
| Topical heparin | 3 (8.3) |

Note: Supportive measures listed were used in ≥5% of patients.

*AE* adverse event; *QW* weekly; *SC* subcutaneous.
^a^With 2–3 step-up doses. ^b^Including dysgeusia, ageusia, hypogeusia, and taste disorder. ^c^Day of AE onset relative to initial step-up dose. ^d^Including AEs with both start and end dates available. ^e^Patients could receive more than one supportive therapy. ^f^Including skin exfoliation, dry skin, pruritus, and palmar-plantar erythrodysesthesia syndrome. ^g^Including nail discoloration, nail disorder, onycholysis, onychomadesis, onychoclasis, nail dystrophy, nail toxicity, and nail ridging. ^h^Including rash, maculopapular rash, erythematous rash, and erythema.
